# ZBTB20 regulates EGFR expression and hepatocyte proliferation in mouse liver regeneration

**DOI:** 10.1038/s41419-018-0514-0

**Published:** 2018-04-27

**Authors:** Hai Zhang, Jian-Hui Shi, Hui Jiang, Kejia Wang, Jun-Yu Lu, Xuchao Jiang, Xianhua Ma, Yu-Xia Chen, An-Jing Ren, Jianming Zheng, Zhifang Xie, Shaodong Guo, Xiongfei Xu, Weiping J. Zhang

**Affiliations:** 1 Department of Pathophysiology, Second Military Medical University, Shanghai, 200433 China; 2 Department of Pathology, Changhai Hospital, Shanghai, 200433 China; 3 Department of Nutrition and Metabolism, Texas University of Agriculture and Mechanics, College Station, TX 77843 USA

## Abstract

Liver has a unique regenerative capacity, however, its regulatory mechanism is not fully defined. We have established the zinc-finger protein ZBTB20 as a key transcriptional repressor for alpha-fetoprotein (AFP) gene in liver. As a marker of hepatic differentiation, AFP expression is closely associated with hepatocyte proliferation. Unexpectedly, here we showed that ZBTB20 acts as a positive regulator of hepatic replication and is required for efficient liver regeneration. The mice specifically lacking ZBTB20 in hepatocytes exhibited a remarkable defect in liver regeneration after partial hepatectomy, which was characterized by impaired hepatocyte proliferation along with delayed cyclin D1 induction and diminished AKT activation. Furthermore, we found that epithelial growth factor receptor (EGFR) expression was dramatically reduced in the liver in the absence of ZBTB20, thereby substantially attenuating the activation of EGFR signaling pathway in regenerating liver. Adenovirus-mediated EGFR overexpression in ZBTB20-deficient hepatocytes could largely restore AKT activation in response to EGFR ligands in vitro, as well as hepatocyte replication in liver regeneration. Furthermore, ZBTB20 overexpression could significantly restore hepatic EGFR expression and cell proliferation after hepatectomy in ZBTB20-deficient liver. Taken together, our data point to ZBTB20 as a critical regulator of EGFR expression and hepatocyte proliferation in mouse liver regeneration, and may serve as a potential therapeutic target in clinical settings of liver regeneration.

## Introduction

The liver is a unique organ with remarkable regenerative capacity. Although adult hepatocytes are quiescent and highly differentiated, they retain the ability to proliferate and regenerate damaged liver tissue in response to toxic injury or infections. Abnormal liver regeneration contributes to the pathogenesis of fulminant liver failure, cirrhosis, and primary liver cancer. Two-thirds partial hepatectomy (PH) is the most studied model of liver regeneration^1,2^. This operation induces almost all hepatocytes to synchronously exit from their quiescent state, rapidly re-enter the cell cycle, and undergo one to two rounds of replication, leading to full restoration of liver mass in 7–10 days in rodents.

Liver regeneration after PH is a complex process, which involves the activation of multiple pathways in a highly orchestrated manner. During the initial priming phase, the G0/G1 transition of hepatocytes is largely regulated by cytokines such as tumor necrosis factor-α (TNF-α) and interleukin-6 (IL-6)^3,4^, which are mainly produced by activated non-parenchymal liver cells (NPCs), for example, Kupffer cells^5^. IL-6 expression and resultant activation of signal transducer and activator of transcription 3 (STAT3) are dependent of TNFR1-mediated nuclear factor κ-B (NF-κB) activation^6,7^, and play an essential role in the induction of immediate early response genes (*c-Fos, c-Jun, Jun-B*, and *c-Myc)* and cyclin D1 expression (entry of G1 phase)^3,8^. Interestingly, PH-stimulated NF-κB activation mainly occurs in NPC during priming phase^9^, and hepatocyte-specific inhibition of NF-κB does not compromise IL-6 production or liver regeneration^10^. During the progression phase, hepatocyte replication is regulated by growth factors such as hepatocyte growth factor (HGF), and epidermal growth factor (EGF) family members such as EGF, transforming growth factor-α (TGF-α), heparin-binding EGF, and amphiregulin (AR). These growth factors are increased at the expression levels after PH, and can stimulate hepatocyte proliferation in vitro mainly through mitogen-activated protein kinase kinase (MEK)/extracellular signal-regulated kinase (ERK) and phosphatidylinositide 3-kinase (PI3K)/protein kinase B (AKT) signaling cascades^1,11–15^. The studies using liver-specific knockout mice demonstrate that HGF receptor (HGFR) signaling is essential for ERK1/2 activation, as well as full AKT activation and liver regeneration, which may primarily affect hepatocyte survival and tissue remodeling^16^, whereas epithelial growth factor receptor (EGFR) pathway regulates hepatocyte proliferation and efficient liver regeneration^17^. Therefore, the expression and activity of HGFR and EGFR play critical roles in liver regeneration. Although the expression of EGFR ligands such as TGF-α are highly regulated by TNF-α and HGF in regenerating liver^4,18^, little is known about the regulation of EGFR expression itself in hepatocytes. In addition, the signaling pathways mediating the mitogenic response of EGFR in regenerating liver has not been clarified.

ZBTB20 (also named as DPZF, ZNF288, and HOF), a new member of BTB/POZ zinc-finger family^19^, plays a variety of important roles in multiple systems^20–28^. In liver, ZBTB20 acts as a key transcriptional repressor mediating postnatal repression of alpha-fetoprotein gene (*Afp*)^29,30^. ZBTB20 deficiency causes a dramatic increase in AFP expression levels^29^, which is consistent with the observations that dysregulated expression or mutation of ZBTB20 leads to AFP reactivation in human^31,32^. More interestingly, ZBTB20 expression is increased in human hepatocellular carcinoma (HCC) and associated with poor prognosis^33,34^, the underlying mechanism of which remains unclear. These prompt us to explore its potential role of ZBTB20 in hepatocyte proliferation. In this study, we show that deletion of hepatic ZBTB20 leads to a delay in liver regeneration due to impaired hepatocyte proliferation. Furthermore, we demonstrate that EGFR expression is substantially reduced in Zbtb20-deficient hepatocytes. As a result, EGFR ligand-induced AKT activation is impaired in both primary hepatocytes and regenerating liver in the absence of ZBTB20. Thus, our findings for the first time establish a critical role of ZBTB20 in hepatic EGFR expression and liver regeneration.

## Results

### Liver regeneration is delayed in the absence of Zbtb20

We have previously generated liver-specific Zbtb20 knockout mice (hereafter LZB20KO)^29^ by crossing mice harboring LoxP-flanked *Zbtb20* allele with albumin-Cre mouse strain. This Cre line mediates efficient recombination approximately 3 weeks after birth^35^, when liver development is already completed. We have shown that LZB20KO mice survive equally into adulthood with no gross abnormalities compared with control, with the liver apparently normal in appearance, size, and architecture. Of note, adult Zbtb20-null hepatocytes are under normally resting status despite aberrant expression of AFP^29^. These suggest that deletion of Zbtb20 in postnatal hepatocytes does not compromise liver development.

**Fig. 1:**
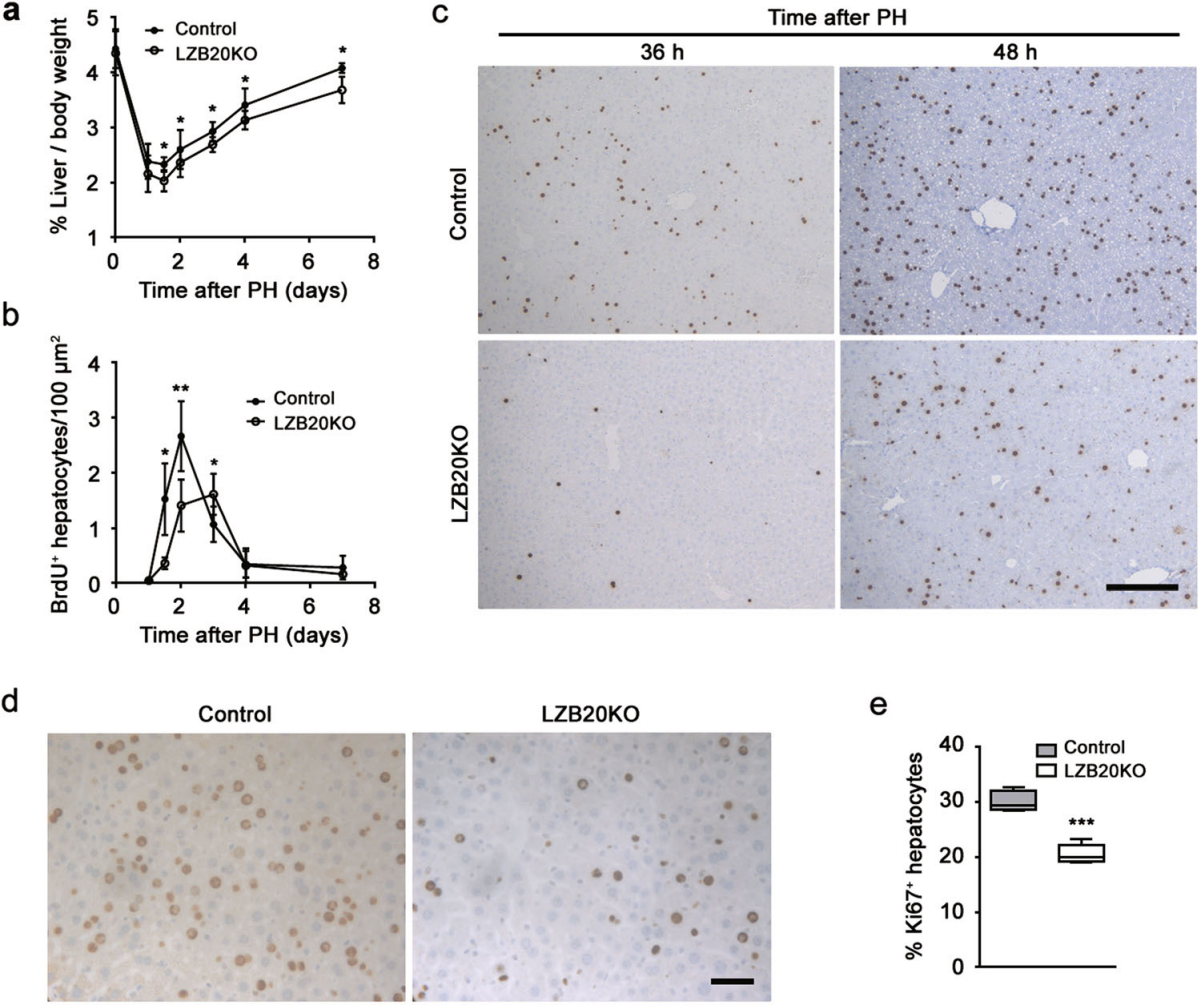
ZBTB20 is required for hepatocyte proliferation in liver regeneration. **a, b** Liver/body weight ratios **a** and hepatic BrdU incorporation **b** of control and LZB20KO mice at indicated time points after PH. *n* = 4~8. BrdU was i.p. injected 2 h before animal sacrifice. Results are represented by the mean ± SD. *P < 0.05; **P < 0.01 versus control (Mann–Whitney test). **c** Representative photographs of BrdU staining on liver sections at 36 and 48 h after PH. *n* = 5. Scale bar, 200 μm. **d, e** Immunostaining **d** and quantification **e** of Ki67-positive hepatocytes on liver sections at 48 h after PH. *n* = 5. Scale bar, 50 μm. The data are presented as box-and-whisker plots, where the line in the box indicates the median, the box extends from the 25th to 75th percentiles, and the whiskers indicate the minimal and maximal values. ***P < 0.001 versus control (Mann–Whitney test)

To evaluate the role of ZBTB20 in liver regeneration, we performed two-thirds PH on LZB20KO and control mice, and subsequently measured the restoration of liver mass. No mortality was observed in either of the two groups within 2 weeks after liver resection. Interestingly, LZB20KO mice exhibited a significant decrease in the liver-to-body weight ratio between 1 and 7 days after PH compared with control, which implied the impairment of liver regeneration (Fig. 1a). However, at 14 days after PH, there was no significant difference in the liver-to-body weight ratio between the two genotypes, indicating that the liver mass is eventually restored to normal levels in the absence of ZBTB20 (Supplementary Fig.1). Moreover, the liver architecture was comparable between mutant and control mice before and after PH (Supplementary Fig.2). These results suggested that ZBTB20 is required for efficient liver regeneration.

### Zbtb20 deficiency impairs hepatocyte proliferation in regenerating liver

To determine the cellular mechanism by which ZBTB20 regulates liver regeneration, we first examined hepatocyte proliferation after PH. As a measure for DNA replication in S phase, BrdU incorporation assay revealed that hepatic DNA replication was first detected in control liver at 36 h after PH, peaked at 48 h, and declined thereafter, which is in agreement with the published results^36^. In contrast, LZB20KO livers displayed a dramatic reduction in hepatic BrdU incorporation rate at 36 and 48 h after PH (Figs. 1b, c, and Supplementary Fig.3). Consistently, the immunostaining of Ki67, a mitotic marker expressed from the mid-G1 phase to the end of mitosis^37^, revealed that Ki67^+^ proliferating hepatocytes were significantly reduced in mutant mice compared with control at 48 h after PH (Fig. 1d,e). These results indicate that ZBTB20 is essential for hepatocyte proliferation during liver regeneration.

To determine whether ZBTB20 is required for hepatocyte survival during liver regeneration, we detected cell apoptosis by terminal deoxynucleotidyl transferase (TdT)-mediated dUTP nick end labeling (TUNEL) staining. In agreement with the published report^38^, hepatic apoptosis was barely detected in either control or mutant mice within 6–48 h after PH (Supplementary Fig.4), suggesting that Zbtb20 is dispensable for hepatocyte survival in liver regeneration. Therefore, these data indicate that the defect of liver regeneration in the absence of ZBTB20 is primarily due to impaired hepatocyte proliferation rather than increased apoptosis.

### ZBTB20 regulates cell cycle progression in regenerating hepatocytes

**Fig. 2:**
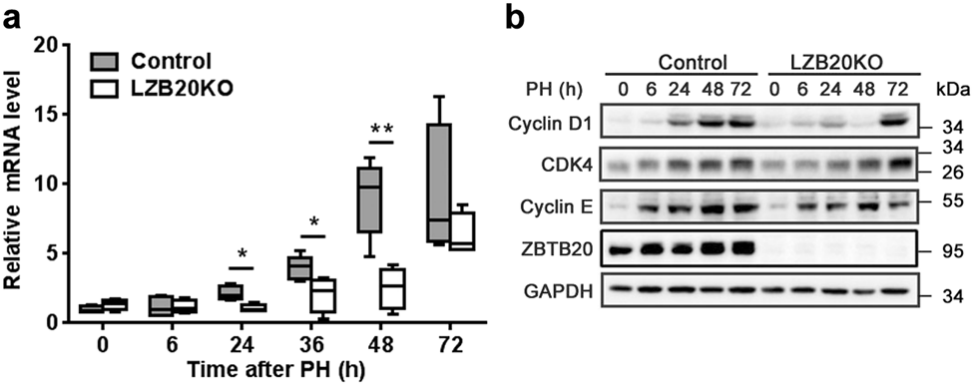
Impaired induction of cyclin D1 in ZBTB20-deficient mice in liver regeneration. **a** qRT-PCR analysis for cyclin D1 mRNA expression in regenerating livers at the indicated time points after PH. **b** Western blot analysis for protein expression of cell cycle regulators in LZB20KO and control liver at the indicated time point after PH. GAPDH was probed as a loading control. The data are presented as box-and-whisker plots. *P < 0.05; **P < 0.01 versus control (Mann–Whitney test); *n* = 4 at each time point

Next, we examined cell cycle progression. PH robustly induces the activation of cyclin D1, a critical regulator of cell cycle progression from G1 to S phase^39^. In control liver, the mRNA levels of cyclin D1 were significantly elevated at 24 h, and maintained at high levels at 48 and 72 h after PH (Fig. 2a). In contrast, the mutant liver displayed a marked delay in the induction of cyclin D1 expression, with the mRNA levels much lower than control at 36 and 48 h after PH. Western blot analyses demonstrated that cyclin D1 protein was significantly induced in control liver at 24 h, and maintained at robust levels at 48 and 72 h after PH, while it was not effectively induced in LZB20KO liver until 72 h after PH (Fig. 2b). However, both mRNA and protein levels of cyclin D1 were comparable in the liver between control and mutant groups at 72 h after PH. The delayed induction of cyclin D1 largely accords with the impairment of hepatic DNA replication in the mutants, whereas the cyclin D1-dependent kinase CDK4 was comparably induced at mRNA and protein levels in both groups. In addition, cyclin E was slightly but significantly reduced at the protein levels in mutant liver compared with control between 6 and 72 h after PH, although its mRNA levels were not affected (Fig. 2b, and Supplementary Fig. 5). Furthermore, ZBTB20 deficiency did not significantly affect the induction of the critical cell cycle regulators such as p21, and p53 in the regenerating liver. Taken together, these findings suggest that ZBTB20 is required for cyclin D1 induction and cell cycle progression in liver regeneration.

### Normal induction of cytokines and early response genes in hepatocyte priming

**Fig. 3:**
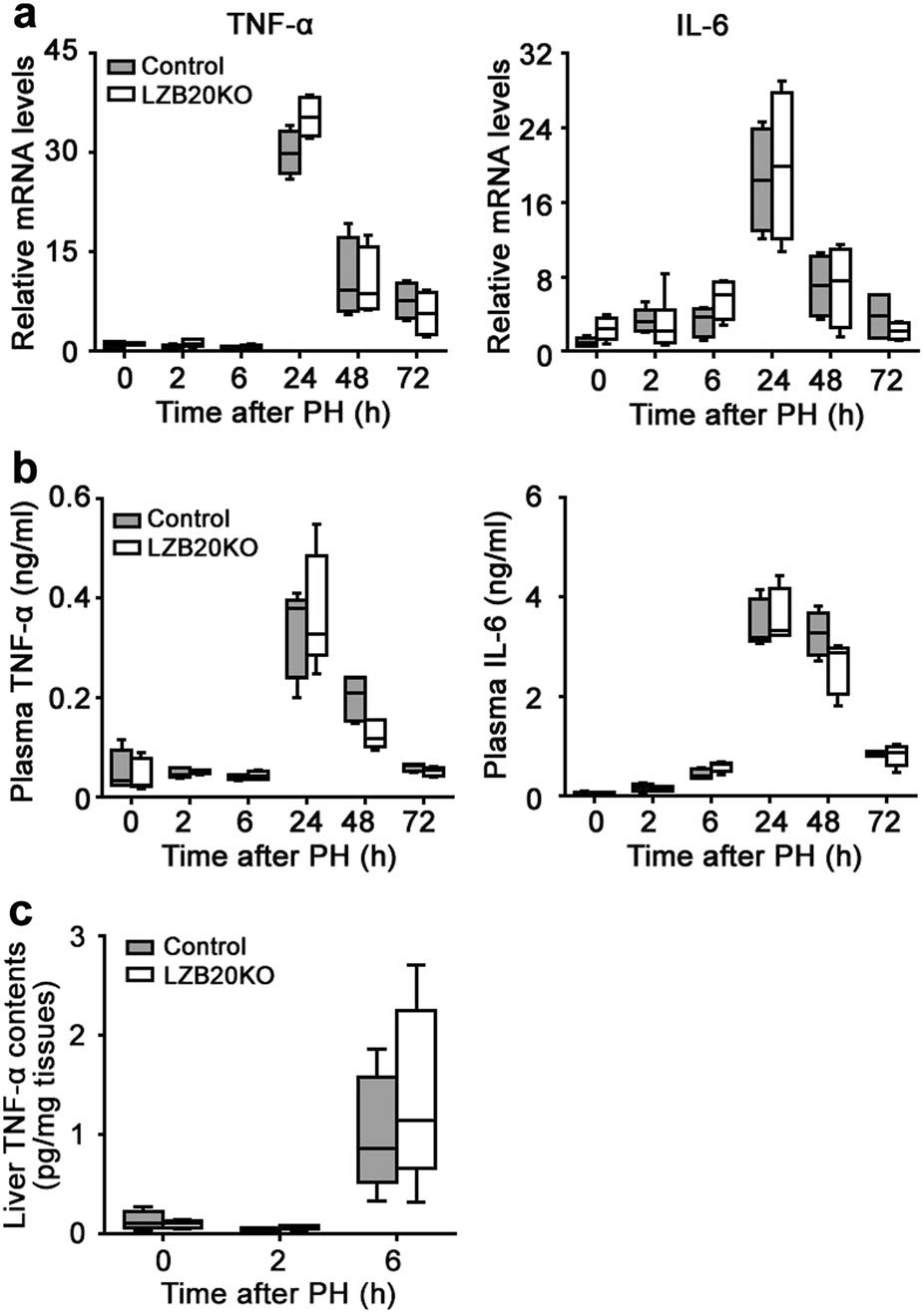
Normal induction of TNF-α and IL-6 in liver regeneration. **a** Quantitative RT-PCR analysis for the mRNA expression of TNF-α and IL-6 in the liver at indicated time points after PH. *n* = 4–6. **b** Plasma contents of TNF-α and IL-6 were detected by ELISA at indicated time points after PH. **c** TNF-α contents in the liver extracts at indicated time points after PH. The data are presented as box-and-whisker plots

To explore the molecular mechanisms by which ZBTB20 regulates hepatocyte proliferation in regenerating liver, we first examined the genes, which are relevant for hepatocyte priming in the early phase of liver regeneration. IL-6 and TNF-α play important role in the hepatocyte priming phase^3^. Within the first 6 h after PH, no significant change of TNF-α was detected at liver mRNA or serum protein levels in both genotypes, whereas IL-6 was significantly and comparably increased (Figs. 3a, b). These two cytokines were robustly elevated at liver mRNA and serum protein levels from 24 to 72 h after PH, with no significant difference between control and mutant groups. In the liver extract, both the groups displayed similar increase in TNF-α protein contents at 6 h after PH (Fig. 3c). Furthermore, reverse transcriptase-PCR (RT-PCR) analysis for the immediate early response genes revealed that the two groups had similar mRNA expression levels of *c-Fos, c-Jun, Jun-B*, and *c-Myc* at 2 h after PH (Supplementary Fig. 6). These results suggested that hepatic ZBTB20 is dispensable for the induction of cytokines and early response genes to prime hepatocytes during the early phase of liver regeneration.

### Impaired hepatic EGFR expression and activation in the absence of ZBTB20

**Fig. 4:**
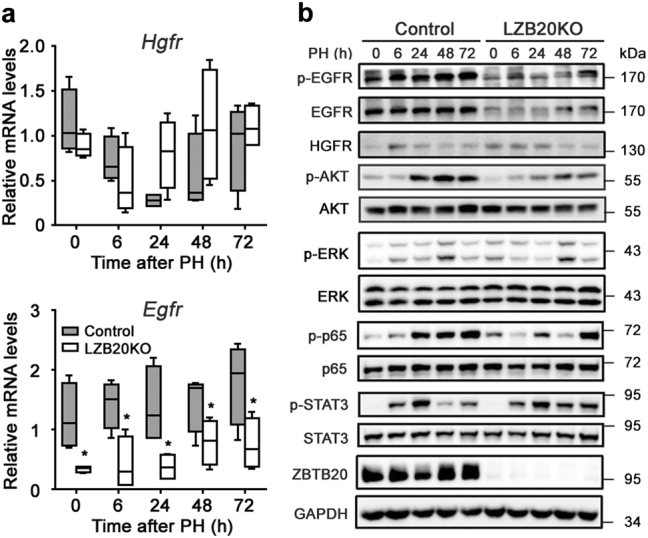
ZBTB20 deficiency impairs EGFR expression and activation in liver regeneration. **a** *Hgfr* and *Egfr* mRNA expression levels were in the mutant liver at the indicated time points after PH. qRT-PCR was performed using 36B4 as internal control. The data are presented as box-and-whisker plots. *P < 0.05 versus control (Mann–Whitney test); *n* = 4–8 at each time point. **b** EGFR protein expression and activation were attenuated in regenerating liver from mutant mice. Western blot was performed on the whole liver lysate with GAPDH as loading control. The activation of EGFR, AKT, and NF-kB was impaired in regenerating liver at the indicated times after PH in the absence of ZBTB20

Next, we examined growth factors and their signaling cascades relevant for hepatocyte cell cycle progression. HGF and EGFR ligands are potent mitogenic factors for hepatocytes in vitro by activating ERK and AKT cascades, and required for liver regeneration^16,17^. HGFR, the signaling of which is essential for ERK activation after PH^16^, was comparably expressed at either mRNA or protein levels in the remnant livers between control and mutant groups (Figs. 4a, b). Interestingly, *Egfr* mRNA levels were reduced by approximately 50–70% in Zbtb20-deficient livers compared with control before or 6–72 h after PH. To a much more extent, EGFR protein levels were substantially decreased in the mutant liver. However, EGFR ligands, such as EGF, HB-EGF, and TGF-α, were comparably expressed at the mRNA levels between both genotypes (Supplementary Fig. 7). Then, we further characterized the activation of relevant signaling pathways in regenerating liver using phosphorylation-specific antibodies. Compared with control, EGFR activation reflected by its tyrosine phosphorylation was severely impaired in mutant from 6 to 72 h after PH (Fig. 4b), which was consistent with its decreased expression levels. Moreover, the activation of AKT and NF-kB was significantly attenuated in mutant, both of which were robustly activated in control from 24 to 72 h after PH, whereas ERK activation was not significantly affected by ZBTB20 deficiency. In consistence with normal induction of IL-6 expression, STAT3 activation was not affected in the regenerating liver by the loss of ZBTB20, which was robustly detected using the antibodies against tyrosine phosphorylated-STAT3 at 6 h, and peaked at 24 h after PH. Collectively, these results suggested that ZBTB20 may play an important role in regulating hepatic EGFR expression and thus its activation to promote hepatocyte proliferation during liver regeneration.

**Fig. 5:**
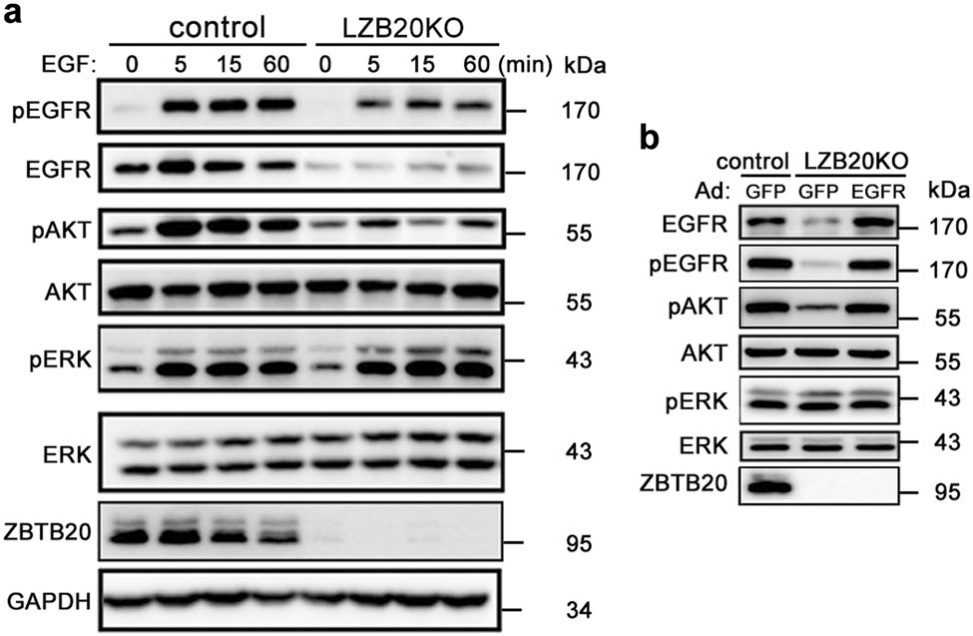
Impaired EGFR signaling pathway in ZBTB20-deficient hepatocytes can be corrected by EGFR overexpression. **a** Primary hepatocytes from LZB20KO and control mice were stimulated with EGF (1 ng/ml) for indicated times following 12 h of serum starvation, and the expression and phosphorylation of EGFR, ERK, and AKT were analyzed by western blot. **b** Hepatocytes were infected with adenoviruses Ad-EGFR or Ad-GFP (MOI = 10) at 24 h prior to 12 h of serum starvation, and followed by EGF stimulation for 5 min. The expression and phosphorylation of EGFR, ERK, and AKT were analyzed by western blot

To further characterize hepatic EGFR signaling pathway in the absence of ZBTB20, we isolated primary hepatocytes from LZB20KO and control mice, and treated them with different growth factors. *Egfr* expression was dramatically reduced in Zbtb20-deficient hepatocytes at protein levels. Treatment of hepatocytes with EGFR ligands such as EGF, HB-EGF, or AR could effectively activate EGFR, ERK, and AKT in control hepatocytes. Of note, the activation of EGFR and AKT was substantially attenuated in ZBTB20-deficient hepatocytes compared with control, whereas ERK activation was not changed (Fig. 5a and Supplementary Fig. 8). However, none of the above EGFR ligands showed activating effect on NF-κB in the hepatocytes of both genotypes (Supplementary Fig. 9). As a positive control, treatment of TNF-α led to the activation of NF-κB in primary hepatocytes, with no significant difference between control and mutant groups. In addition, HGF treatment resulted in a robust activation of AKT and ERK in primary hepatocytes, which was not significantly different between control and mutant groups (Supplementary Fig. 10). These results suggest that ZBTB20 deficiency selectively attenuates the activation of hepatic AKT signaling by EGFR ligands due to reduced EGFR expression. In combined with the critical role of PI3K/AKT pathway in hepatocyte proliferation^11,13,14^, impaired activation of EGFR and its downstream AKT pathway is likely to cause a defect of hepatocyte proliferation in liver regeneration in the absence of ZBTB20.

### EGFR overexpression partially restored EGFR signaling and liver regeneration

**Fig. 6:**
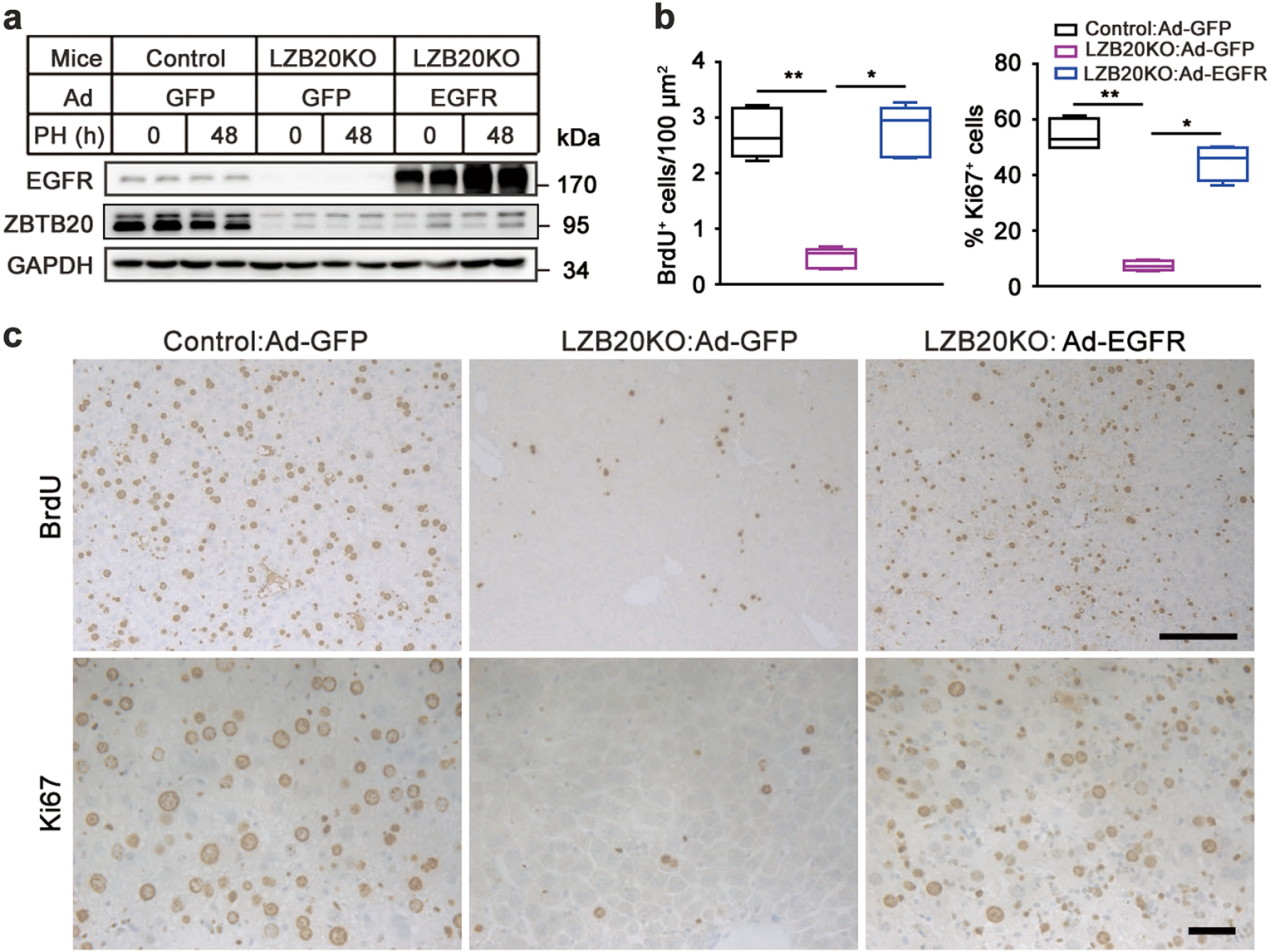
EGFR overexpression restores the impaired hepatocyte proliferation in regenerating liver from Zbtb20-deficient mice. LZB20KO and control mice were i.v. injected with adenoviruses Ad-GFP or Ad-EGFR (0.2 O.D. per mouse) at 48 h prior to PH, and their liver regeneration was analyzed at 48 h after surgeries. BrdU was i.p. injected 2 h before animal sacrifice. **a** EGFR expression in the liver was assayed by western blotting. **b** The frequency of BrdU incorporation and Ki67 expression in hepatocytes 48 h after PH. The data are presented as box-and-whisker plots. *P < 0.05; **P < 0.01 (ANOVA test); *n* = 4–8. **c** Representative photographs for the immunostaining of BrdU and Ki67 on liver sections at 48 h after PH. Scale bar (upper), 200 μm; scale bar (lower), 50 μm

Given the critical role of EGFR in liver regeneration^17^, we reasoned that the impaired EGFR activation might at least partially contribute to the defect of hepatocyte proliferation in the absence of ZBTB20. To address this hypothesis, we first infected ZBTB20-null primary hepatocytes in vitro with recombinant adenoviruses expressing EGFR (Ad-EGFR) or green fluorescent protein (GFP) (Ad-GFP) as control. EGFR overexpression could effectively restore AKT activation by EGF compared with GFP control (Fig. 5b). Then, we further examined the rescuing effects of Ad-EGFR on liver regeneration. Intravenous injection of Ad-EGFR led to EGFR overexpression in LZB20KO liver compared with GFP control (Fig. 6a). Two days after adenoviral administration, PH was performed on the mice, and their liver regeneration was evaluated by hepatocyte proliferation at 48 h after PH. EGFR overexpression could largely correct the defect of hepatocyte replication in LZB20KO mice as reflected by the recovery of BrdU incorporation and Ki67 expression in hepatocytes (Figs. 6b, c). Due to acute liver inflammatory response triggered by adenoviruses themselves, we did not analyze liver/body mass or EGFR signaling pathway after PH in the rescuing experiment. These data suggest that impaired EGFR expression and activation at least partly accounts for the defect of liver regeneration in the absence of ZBTB20.

### ZBTB20 overexpression partially restores EGFR expression and liver regeneration

**Fig. 7:**
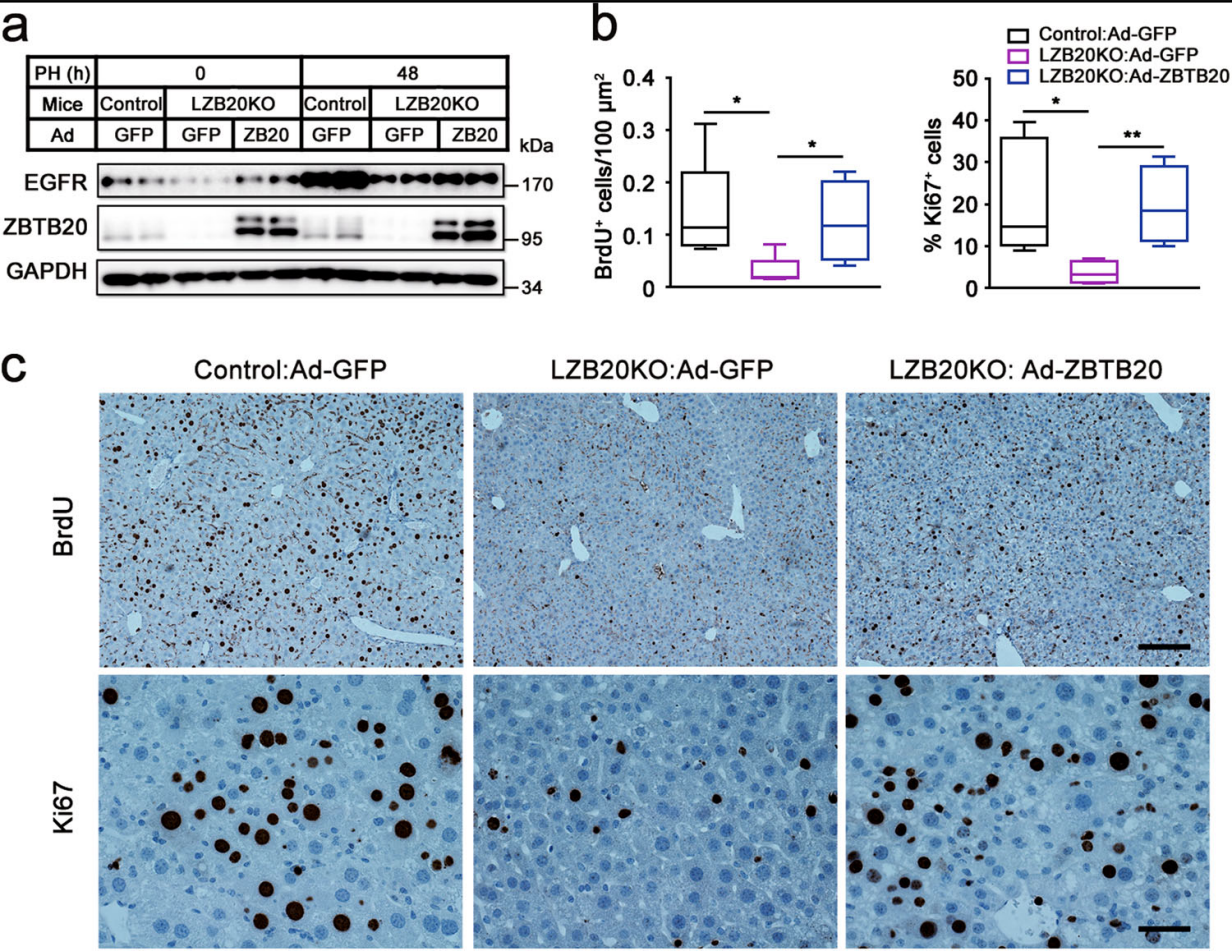
ZBTB20 overexpression restores EGFR expression and hepatocyte proliferation in regenerating liver from Zbtb20-deficient mice. LZB20KO and control mice (3–5 months old) were i.v. injected with adenoviruses Ad-GFP or Ad-ZBTB20 (0.1 O.D. per mouse) 14 days prior to PH, and their liver regeneration was analyzed at 48 h after surgeries. BrdU was i.p. injected 2 h before animal sacrifice. **a** EGFR expression in the liver was assayed by western blotting, and quantified in Supplementary Fig. 11. **b** The frequency of BrdU incorporation and Ki67 expression in hepatocytes at 48 h after PH. The data are presented as box-and-whisker plots. *P < 0.05; **P < 0.01; *n* = 4–6. **c** Representative photographs for the immunostaining of BrdU and Ki67 on liver sections at 48 h after PH. Scale bar (upper), 200 μm; scale bar (lower), 50 μm

To further confirm the regulation of EGFR expression and liver regeneration by ZBTB20, we performed adenovirus-mediated overexpression of ZBTB20 in LZB20KO liver prior to PH. Intravenous administration of adenoviruses expressing ZBTB20 (Ad-ZBTB20) resulted in a robust overexpression of ZBTB20 in ZBTB20-deficient liver through 2 weeks, whereas a mild increase in EGFR expression both at mRNA and protein levels were observed both prior to and at 48 h after PH compared with GFP control (Fig. 7a and Supplementary Fig.11). Remarkably, the defect of hepatocyte replication in LZB20KO mice was largely corrected by ZBTB20 overexpression, as evidenced by the recovery of BrdU incorporation and Ki67 expression in the hepatocytes (Figs. 7b, c). These data strongly suggest that ZBTB20 is capable of regulating both EGFR expression and hepatocyte proliferation in regenerating liver.

### ZBTB20 does not directly regulate Egfr gene transcription

To address whether ZBTB20 acts as a transcription factor to directly regulate *Egfr* gene transcription, we first performed chromatin immunoprecipitation (ChIP) analysis to examine their potential physical binding at chromatin levels. No significant binding of ZBTB20 to *Egfr* gene was detected in the liver using three sets of PCR primers spanning –3-kb upstream the transcriptional start site (Supplementary Fig.12). By contrast, ZBTB20 showed a robust binding to *Afp* promoter as a positive control^29^. Furthermore, luciferase reporter assay showed that ZBTB20 overexpression dramatically repressed *Afp* promoter activity in hepatocellular carcinoma cells HepG2 as a positive control, but had no significant effect on the transcriptional activity of a mouse *Egfr* promoter (spanning 3.7-kb upstream transcriptional start site). These results excluded the possibility that ZBTB20 directly regulates *Egfr* gene transcription. Put together, thus we postulate that ZBTB20 may regulate EGFR expression by an indirect mechanism, thereby modulating the activation of EGFR and its downstream AKT signaling pathway, which at least partly contributes to cyclin D1 induction and hepatocyte proliferation in liver regeneration (Supplementary Fig.13).

## Discussion

In this study, we examined the role of ZBTB20 in liver regeneration. Our findings provide compelling evidence that ZBTB20 regulates hepatic EGFR expression and is required for efficient liver regeneration. First, ZBTB20 is highly expressed in adult differentiated hepatocytes^29^. Second, specific ablation of hepatic Zbtb20 leads to a significant defect of liver regeneration, as manifested by the reduction in the ratios of liver mass to body weight and the impairment in cyclin D1 induction and hepatocyte replication. Third, ZBTB20 deficiency drastically decreases hepatic expression of EGFR, which is required for efficient liver regeneration^17^. As a result, the activation of AKT, a key component in PI3K signaling cascade mediating the mitogenic response of hepatocytes to growth factors^11,13,14^, is diminished in the regenerating liver. Furthermore, EGFR overexpression can at least partially correct the impaired AKT activation and hepatocyte proliferation in liver regeneration. Finally, ZBTB20 overexpression leads to an significant increase in hepatic EGFR expression and subsequent cell replication in regenerating liver. Thus, our data establishes a critical role of ZBTB20 in EGFR expression and hepatocyte proliferation in liver regeneration.

Regarding to liver regeneration, LZB20KO mice are quite similar to the mice lacking hepatic EGFR in terms of the phenotype and mechanism. Both lines show decreased ratios of liver mass to body weight, impaired induction of cyclin D1 and cell cycle progression, diminished activation of NF-κB pathway in regenerating liver, with apparently normal activation of liver ERK1/ERK2 pathway and expression of TNF-α and IL-6^17^. However, there is some discrepancy between the two models. In LZB20KO mice, AKT activation is significantly impaired in both regenerating liver and EGFR ligand-stimulated primary hepatocytes. In contrast, AKT activation seems quite weak but normal in regenerating liver from the mice lacking hepatic EGFR, however, which has not been examined in primary hepatocytes upon stimulation with EGFR ligands^17^. Furthermore, our data show that EGFR overexpression can rescue the defect of AKT activation in Zbtb20-null hepatocytes in vitro. In combined with the critical role of PI3K/AKT pathway in hepatocyte proliferation^11,13,14^, we tend to believe AKT pathway contributes to EGFR-mediated hepatocyte proliferation in liver regeneration. On the other hand, although impaired NF-κB activation was observed in the liver regeneration of both models, whether it occurs in hepatocytes or NPCs needs to be determined. We found that ZBTB20 deficiency did not alter NF-κB activation in primary hepatocytes stimulated with TNF-α. Although EGF can activate non-classical NF-kB signaling pathway via ABIN in tumor cells^40^, our data suggest that EGF-induced NF-κB activation may not occur in primary hepatocytes. Moreover, PH-stimulated NF-κB activation mainly occurs in NPC during priming phase^9^, and hepatocyte-specific inhibition of NF-κB does not compromise IL-6 production or liver regeneration^10^. Therefore, it is unlikely that the defect of cyclin D1 induction and hepatocyte proliferation in both liver-specific knockout mice is attributed to impaired NF-κB activation in hepatocytes. As the expression of TNF-α and IL-6 is not affected, the mechanism and significance of the impairment of NF-κB activation are still not clear in the two models. Meanwhile, we cannot exclude other mechanisms beyond EGFR by which ZBTB20 regulates liver regeneration. It is noteworthy that ZBTB20 also regulates de novo lipogenesis in liver^41^. Whether ZBTB20 regulated lipogenesis is involved in liver regeneration needs further investigation.

The finding that ZBTB20 promotes hepatocyte proliferation in liver regeneration is consistent with our previous observation revealing its increased expression and negative prognostic significance in HCC^33,34^. It is an intriguing subject whether ZBTB20/EGFR pathway plays a role in hepatocellular carcinogenesis, which is under further investigation. On the other hand, given the positive close association between AFP expression and hepatocyte proliferation^42,43^, we had expected that LZB20KO mice might show an advantage in liver regeneration in the presence of dysregulated AFP expression. In contrast, our data indicate that ZBTB20 promotes hepatocyte replication while repressing AFP gene transcription, which suggests that AFP expression and hepatocyte proliferation can be mutually independent events, at least in the absence of ZBTB20. Meanwhile, there is still another concern that aberrantly expressed AFP itself may affect liver regeneration in the mice lacking ZBTB20. Given that high level serum AFP is associated with fast-growing and poorly differentiated HCC, it has been postulated that AFP might possess some growth regulatory properties rather than only a serum tumor marker^44^. However, immunotherapy against AFP reveals that AFP does not have a role in liver regeneration^45,46^. In this study, we also found that genetic deletion of hepatic AFP did not alter the liver regeneration phenotype of LZB20KO mice (data not shown). Therefore, these data suggest that ZBTB20 regulates liver regeneration in an AFP-independent manner.

Taken together, this study establishes a critical role of ZBTB20 in hepatocyte biology and liver regeneration, and provides novel insights into the regulation of hepatocyte proliferation and its relationship with AFP.

## Materials and methods

### Animal model and PH

Liver-specific ZBTB20 knockout mice were generated by crossing ZBTB20^flox/flox^ mice with albumin-Cre transgenic mice as previously described^29^. Mice were maintained on chow diet in a specific pathogen free (SPF) facility. Littermates carrying the loxP-flanked alleles but lacking expression of Cre recombinase were used as wild-type controls. Animal experiments were done following institutional guidelines and with approved protocols. Male mice at the age of 8–12 weeks were subjected to PH under isoflurane anesthesia as described^47^. The left lateral lobe and median lobe were ligated and removed. The gall bladder was always removed during surgery to avoid its damage. In the rescuing experiments, PH was performed at 2 weeks after i.v. administration by tail vein with recombinant adenoviruses expressing human EGFR (Ad-EGFR), expressing human ZBTB20 (Ad-ZBTB20), or expressing GFP as control (Ad-GFP) with the dose of 0.1 or 0.2 O.D. per mouse, respectively. The recombinant adenoviruses were purified by CsCl-gradient ultracentrifugation and titrated as previously described^27^.

### Primary hepatocytes isolation and culture

Primary hepatocytes were isolated by collagenase perfusion as described previously^22^. Hepatocytes were stimulated by 20 ng/ml HGF (Peprotech), 10 ng/ml TNF-α (Peprotech), or EGFR ligands such as 1 ng/ml EGF (Peprotech), 1 ng/ml HB-EGF (Peprotech), and 10 ng/ml amphiregulin (Peprotech) for various minutes after serum starvation of 12 h. For adenovirus-mediated gene transfection, hepatocytes were cultured in the presence of adenoviruses with the multiplicity of infection (MOI) of 100 for 6 h, and refreshed with normal media later. Twenty-four hours after adenovirus infection, the hepatocytes were subjected to serum starvation prior to the stimulation with growth factors as described above.

### Luciferase reporter assay

The luciferase reporter plasmids for mouse *Egfr* promoter (–3.7 kb) (#437) cloned from mouse genomic DNA by PCR, and inserted into pGL4.1 vector (Promega). The –1kb mouse Afp promoter-driven luciferase reporter plasmids (#110) and human ZBTB20 expression plasmid have been described previously^29^. Primary hepatocytes or HepG2 cells were seeded in a 24-well plate (1.5 × 10^5^ per well) and transfected with plasmids by Lipofectamine 3000 (Invitrogen). After 48 h of transfection, cells were harvested and disrupted, and the luciferase activities were analyzed using the dual luciferase assay kit (Promega) and normalized against the activity of the Renilla luciferase as internal control.

### mRNA expression analysis

Total RNA was isolated from liver or hepatocytes by Trizol (Invitrogen) reagent. Real-time RT-PCR analysis for mRNA was performed in a two-step reaction. First-strand complementary DNA was synthesized with a reverse transcription kit (Invitrogen), and the second step was performed in a fluorescent temperature cycler (Mastercycler ep realplex, Eppendorf) with SYBR green and specific primers for each of the genes. Every plate included the 36B4 gene as an internal control. The primer sequences are available on request. Results were analyzed with the Student’s unpaired *t-*test. The expression of gene was calculated with the 2^-(△△Ct)^ method.

### Immunoblotting and immunohistochemistry

Protein lysates were prepared in liver homogenization buffer^48^ or RIPA buffer containing 0.5 mM phenylmethane sulfonyl fluoride (PMSF), 4 μg/ml leupeptin, 4 μg/ml aprotinin, and 4 μg/ml pepstatin, separated by sodium dodecyl sulfate (SDS)-polyacrylamide gel electrophoresis (PAGE), transferred to polyvinylidene fluoride (PVDF) membranes, and analyzed by immunoblotting. Antibodies were listed in Supplementary Table 1. Mice were injected i.p. with BrdU (Sigma) at 100 µg/g of body weight 2 h before sacrifice. Liver tissues were fixed overnight in 4% paraformaldehyde, embedded in paraffin, and used for H&E staining, as well as immunostaining with antibodies against BrdU (BD Bioscience) and against Ki67 antibodies (Dako), respectively. The immunostaining was finally developed with diaminobenzidine (DAB). To quantify hepatocyte proliferation, five fields were randomly chosen under microscope per slide after immunostaining to count BrdU or Ki67-positive hepatocytes, and the frequency of BrdU-positive hepatocytes were calculated against the area size of the fields (μm^2^), while the percentage of Ki67-positive hepatocytes was calculated against total hepatocytes in the fields.

### Cytokine detection

The plasma in blood was collected at various times after PH. Then, the cytokines in the plasma including TNF-α and IL-6 were assayed by enzyme-linked immunosorbent assay (ELISA) kits (R&D Systems) according to the manufacturer’s protocols. To detect TNF-α contents in liver extracts, liver tissues (about 20 mg per liver) were homogenized with an automatic homogenizer in 0.5 ml ice-cold phosphate-buffered saline containing 0.1% Tween 20 and 0.5 mM PMSF, and the supernatants were harvested after centrifugation (12,000 *g*, 4 ℃) for 15 min.

### ChIP assay

Liver tissue was isolated from normal adult mice and flash-frozen in liquid nitrogen. ChIP assays of liver tissue were performed as described^27^, with minimal modifications. The chromatin was fragmented by sonication to approximately 300–1000 bp in size. After pre-clearance, the chromatin lysate was incubated overnight with specific antibodies for ChIP such as anti-histone H3 (Abcam), anti-ZBTB20, and normal rabbit IgG (Upstate). To analyze specific, antibody- and protein-bound DNA, conventional PCR and quantitative real-time PCR were performed. Three sets of PCR primers were picked up to amplify the *Egfr* promoter, respectively, the products of which are overlapped each other and cover from its transcription start site to upstream 3 kb. The primer sequences are available on request.

### Statistics

Data are presented as mean ± standard deviation of the mean (SD). Statistical significance between experimental groups was assessed using Mann–Whitney test. Statistical significance among more than two groups was assessed using ANOVA. Significance was accepted at least at the level of *P* < 0.05.

## Electronic supplementary material

Supplemental material

## References

[1] Taub R (2004). Liver regeneration: from myth to mechanism. Nat. Rev. Mol. Cell. Biol..

[2] Michalopoulos GK (2010). Liver regeneration after partial hepatectomy: critical analysis of mechanistic dilemmas. Am. J. Pathol..

[3] Cressman DE (1996). Liver failure and defective hepatocyte regeneration in interleukin-6-deficient mice. Science.

[4] Gallucci RM, Simeonova PP, Toriumi W, Luster MI (2000). TNF-alpha regulates transforming growth factor-alpha expression in regenerating murine liver and isolated hepatocytes. J. Immunol..

[5] Aldeguer X (2002). Interleukin-6 from intrahepatic cells of bone marrow origin is required for normal murine liver regeneration. Hepatology.

[6] Li W, Liang X, Kellendonk C, Poli V, Taub R (2002). STAT3 contributes to the mitogenic response of hepatocytes during liver regeneration. J. Biol. Chem..

[7] Yamada Y, Kirillova I, Peschon JJ, Fausto N (1997). Initiation of liver growth by tumor necrosis factor: deficient liver regeneration in mice lacking type I tumor necrosis factor receptor. Proc. Natl. Acad. Sci. USA.

[8] Wuestefeld T (2003). Interleukin-6/glycoprotein 130-dependent pathways are protective during liver regeneration. J. Biol. Chem..

[9] Seki E (2005). Contribution of Toll-like receptor/myeloid differentiation factor 88 signaling to murine liver regeneration. Hepatology.

[10] Chaisson ML, Brooling JT, Ladiges W, Tsai S, Fausto N (2002). Hepatocyte-specific inhibition of NF-kappaB leads to apoptosis after TNF treatment, but not after partial hepatectomy. J. Clin. Invest..

[11] Band CJ, Mounier C, Posner BI (1999). Epidermal growth factor and insulin-induced deoxyribonucleic acid synthesis in primary rat hepatocytes is phosphatidylinositol 3-kinase dependent and dissociated from protooncogene induction. Endocrinology.

[12] Talarmin H (1999). The mitogen-activated protein kinase kinase/extracellular signal-regulated kinase cascade activation is a key signalling pathway involved in the regulation of G(1) phase progression in proliferating hepatocytes. Mol. Cell. Biol..

[13] Coutant A (2002). PI3K-FRAP/mTOR pathway is critical for hepatocyte proliferation whereas MEK/ERK supports both proliferation and survival. Hepatology.

[14] Berasain C (2005). Amphiregulin: an early trigger of liver regeneration in mice. Gastroenterology.

[15] Mitchell C (2005). Heparin-binding epidermal growth factor-like growth factor links hepatocyte priming with cell cycle progression during liver regeneration. J. Biol. Chem..

[16] Borowiak M (2004). Met provides essential signals for liver regeneration. Proc. Natl. Acad. Sci. USA.

[17] Natarajan A, Wagner B, Sibilia M (2007). The EGF receptor is required for efficient liver regeneration. Proc. Natl. Acad. Sci. USA.

[18] Tomiya T (2000). The mitogenic activity of hepatocyte growth factor on rat hepatocytes is dependent upon endogenous transforming growth factor-alpha. Am. J. Pathol..

[19] Zhang W (2001). Identification and characterization of DPZF, a novel human BTB/POZ zinc finger protein sharing homology to BCL-6. Biochem. Biophys. Res. Commun..

[20] Sutherland AP (2009). Zinc finger protein Zbtb20 is essential for postnatal survival and glucose homeostasis. Mol. Cell. Biol..

[21] Xie Z (2010). Zbtb20 is essential for the specification of CA1 field identity in the developing hippocampus. Proc. Natl. Acad. Sci. USA.

[22] Zhang Y (2012). The zinc finger protein ZBTB20 regulates transcription of fructose-1,6-bisphosphatase 1 and beta cell function in mice. Gastroenterology.

[23] Liu X (2013). Zinc finger protein ZBTB20 promotes Toll-like receptor-triggered innate immune responses by repressing IkappaBalpha gene transcription. Proc. Natl. Acad. Sci. USA.

[24] Ren AJ (2014). ZBTB20 regulates nociception and pain sensation by modulating TRP channel expression in nociceptive sensory neurons. Nat. Commun..

[25] Zhou G (2015). Zbtb20 regulates the terminal differentiation of hypertrophic chondrocytes via repression of Sox9. Development.

[26] Nagao M, Ogata T, Sawada Y, Gotoh Y (2016). Zbtb20 promotes astrocytogenesis during neocortical development. Nat. Commun..

[27] Cao D (2016). ZBTB20 is required for anterior pituitary development and lactotrope specification. Nat. Commun..

[28] Qu Z (2016). Loss of ZBTB20 impairs circadian output and leads to unimodal behavioral rhythms. Elife.

[29] Xie Z (2008). Zinc finger protein ZBTB20 is a key repressor of alpha-fetoprotein gene transcription in liver. Proc. Natl. Acad. Sci. USA.

[30] Zhang H (2015). ZBTB20 is a sequence-specific transcriptional repressor of alpha-fetoprotein gene. Sci. Rep..

[31] Kojima K (2011). MicroRNA122 is a key regulator of alpha-fetoprotein expression and influences the aggressiveness of hepatocellular carcinoma. Nat. Commun..

[32] Cordeddu V (2014). Mutations in ZBTB20 cause Primrose syndrome. Nat. Genet..

[33] Wang Q (2011). Zinc finger protein ZBTB20 expression is increased in hepatocellular carcinoma and associated with poor prognosis. Bmc. Cancer.

[34] Kan H (2016). Zinc finger protein ZBTB20 is an independent prognostic marker and promotes tumor growth of human hepatocellular carcinoma by repressing FoxO1. Oncotarget.

[35] Postic C, Magnuson MA (2000). DNA excision in liver by an albumin-Cre transgene occurs progressively with age. Genesis.

[36] Behrens A (2002). Impaired postnatal hepatocyte proliferation and liver regeneration in mice lacking c-jun in the liver. EMBO J..

[37] Scholzen T, Gerdes J (2000). The Ki-67 protein: from the known and the unknown. J. Cell. Physiol..

[38] Sakamoto T (1999). Mitosis and apoptosis in the liver of interleukin-6-deficient mice after partial hepatectomy. Hepatology.

[39] Nelsen CJ, Rickheim DG, Timchenko NA, Stanley MW, Albrecht JH (2001). Transient expression of cyclin D1 is sufficient to promote hepatocyte replication and liver growth in vivo. Cancer Res..

[40] Huang L (2008). ABINs inhibit EGF receptor-mediated NF-kappaB activation and growth of EGF receptor-overexpressing tumour cells. Oncogene.

[41] Liu G (2017). Regulation of hepatic lipogenesis by the zinc finger protein Zbtb20. Nat. Commun..

[42] Spear BT (1999). Alpha-fetoprotein gene regulation: lessons from transgenic mice. Semin. Cancer Biol..

[43] Peterson ML, Ma C, Spear BT (2011). Zhx2 and Zbtb20: novel regulators of postnatal alpha-fetoprotein repression and their potential role in gene reactivation during liver cancer. Semin. Cancer Biol..

[44] Mizejewskia GJ, Butterstein G (2006). Survey of functional activities of alpha-fetoprotein derived growth inhibitory peptides: review and prospects. Curr. Protein Pept. Sci..

[45] Geissler M (2001). Immunotherapy directed against alpha-fetoprotein results in autoimmune liver disease during liver regeneration in mice. Gastroenterology.

[46] Hanke P, Serwe M, Dombrowski F, Sauerbruch T, Caselmann WH (2002). DNA vaccination with AFP-encoding plasmid DNA prevents growth of subcutaneous AFP-expressing tumors and does not interfere with liver regeneration in mice. Cancer Gene. Ther..

[47] Mitchell C, Willenbring H (2008). A reproducible and well-tolerated method for 2/3 partial hepatectomy in mice. Nat. Protoc..

[48] Guo S (2009). The Irs1 branch of the insulin signaling cascade plays a dominant role in hepatic nutrient homeostasis. Mol. Cell. Biol..

