# Supplementary material for: ZBTB20 regulates EGFR expression and hepatocyte proliferation in mouse liver regeneration
Source: Cell Death Dis. 2018 Apr 27;9(5):462. doi: 10.1038/s41419-018-0514-0 (PMC5920068; doi:10.1038/s41419-018-0514-0)
Supplement: Supplementary file 1 — Supplemental material [file 41419_2018_514_MOESM1_ESM.pdf]

Supplementary Table 1. Antibody list

Antibody	Company	Category #
anti-EGFR	Cell signaling Technology	#4267
anti-pEGFR	Cell signaling Technology	#3777
anti-HGFR	Cell signaling Technology	#8198
anti-AKT	Cell signaling Technology	#9272
anti-pAKT	Cell signaling Technology	#4060
anti-ERK	Cell signaling Technology	#4695
anti-pERK1/ERK2	Cell signaling Technology	#4370
anti-NF-κB p65	Cell signaling Technology	#8242
anti-NF-κB phos-p65	Cell signaling Technology	#3033
anti-Cyclin D1	Cell signaling Technology	#2978
anti-CDK4	Cell signaling Technology	#2906
anti-Cyclin E	Santa Cruz Biotechnology	sc-481
anti-STAT3	Cell signaling Technology	#9139
anti-p-STAT3	Cell signaling Technology	#9131
anti-BrdU	BD Bioscience	550803
anti-Ki67	Abcam	Ab16667

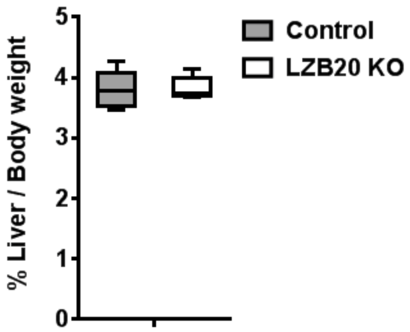

Supplementary Fig.1. **Liver mass is normally restored at 14 days after partial hepatectomy in the absence of ZBTB20**. The data are presented as box-and-whisker plots. n=5-7.

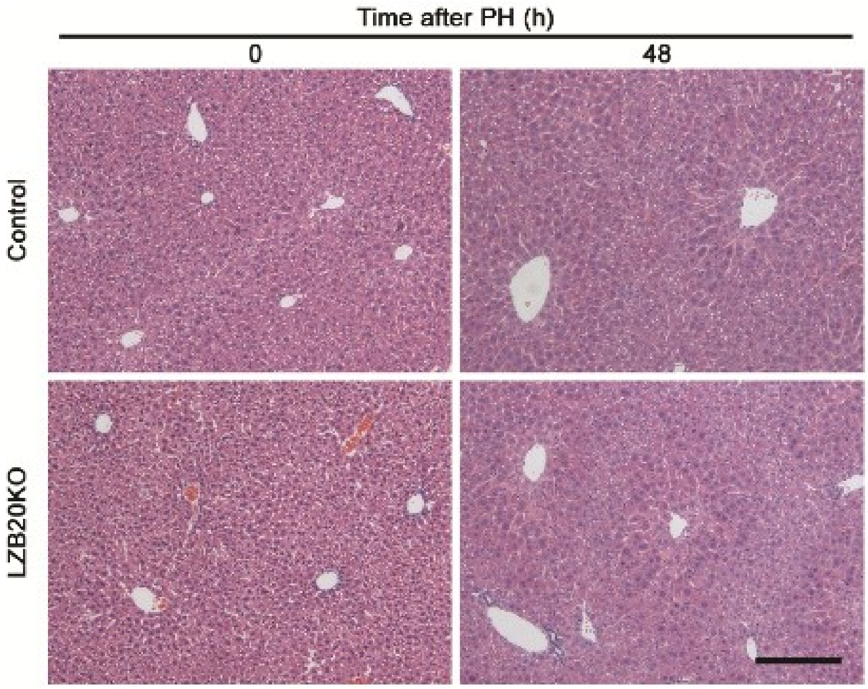

Supplementary Fig.2. HE staining reveals normal architecture in regenerating liver from LZB20KO mice 48 h after partial hepatectomy. Scale bar, 100 μm.

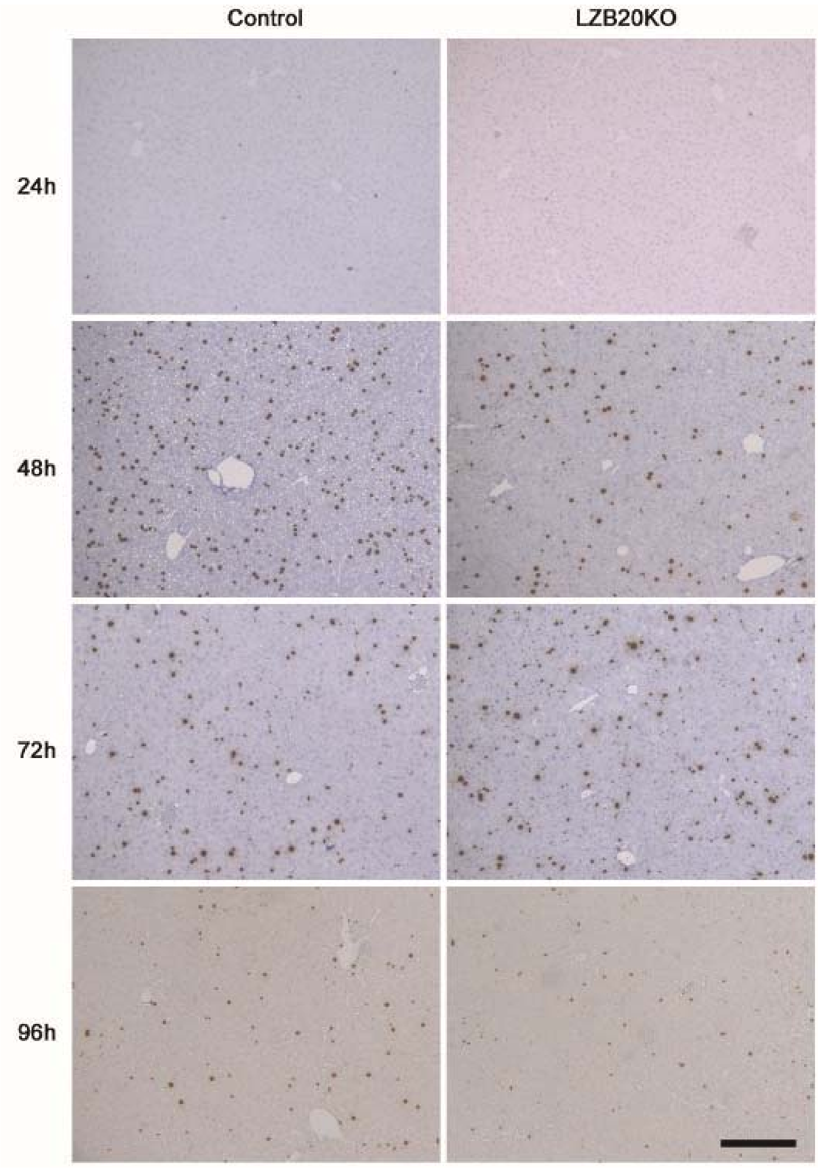

Supplementary Fig.3. **Decreased hepatocyte proliferation in liver regeneration at indicated time points after PH in the absence of Zbtb20.** Representative photographs of BrdU staining on liver sections at indicated time points after PH. n=5~6. Scale bar, 200 μm.

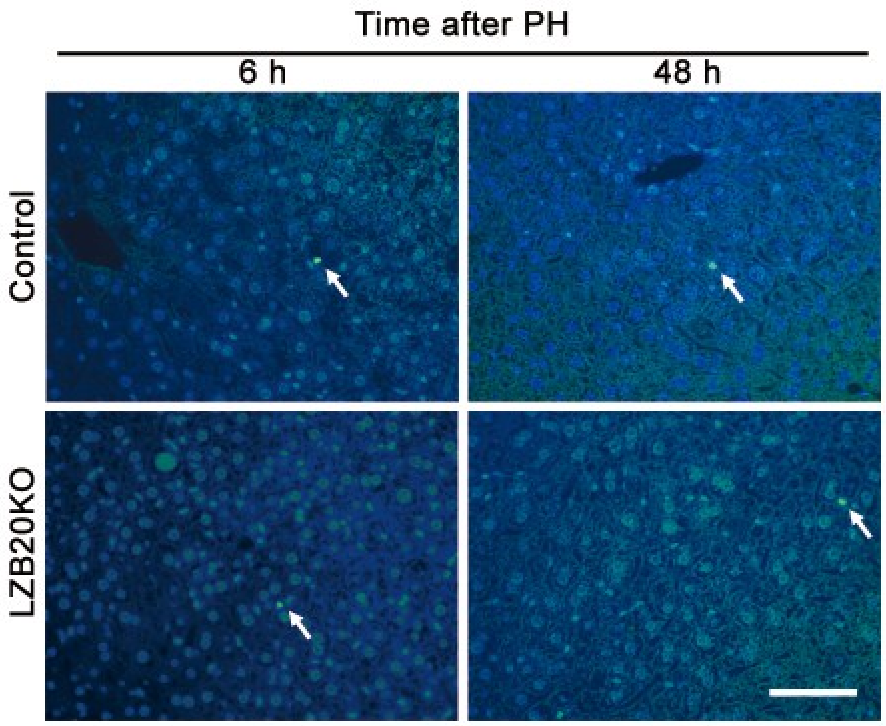

Supplementary Fig.4. **TUNEL staining reveals unaffected cell apoptosis in the regenerating liver in the absence of Zbtb20**. Apoptotic cells are rare at the indicated time after PH. The while arrows indicate apoptotic cells. Representative photos for the results from 4 mice per group. Scale bar, 50 μm.

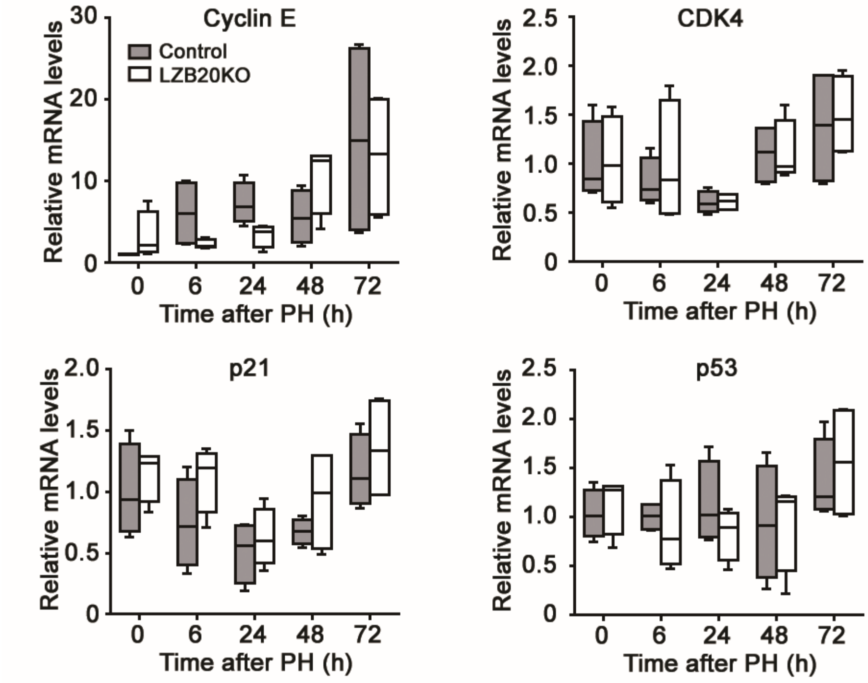

Supplementary Fig.5. **RT-PCR analysis for mRNA expression of the cell cycle regulators at indicated time points after partial hepatectomy**. The data are presented as box-and-whisker plots. n=4.

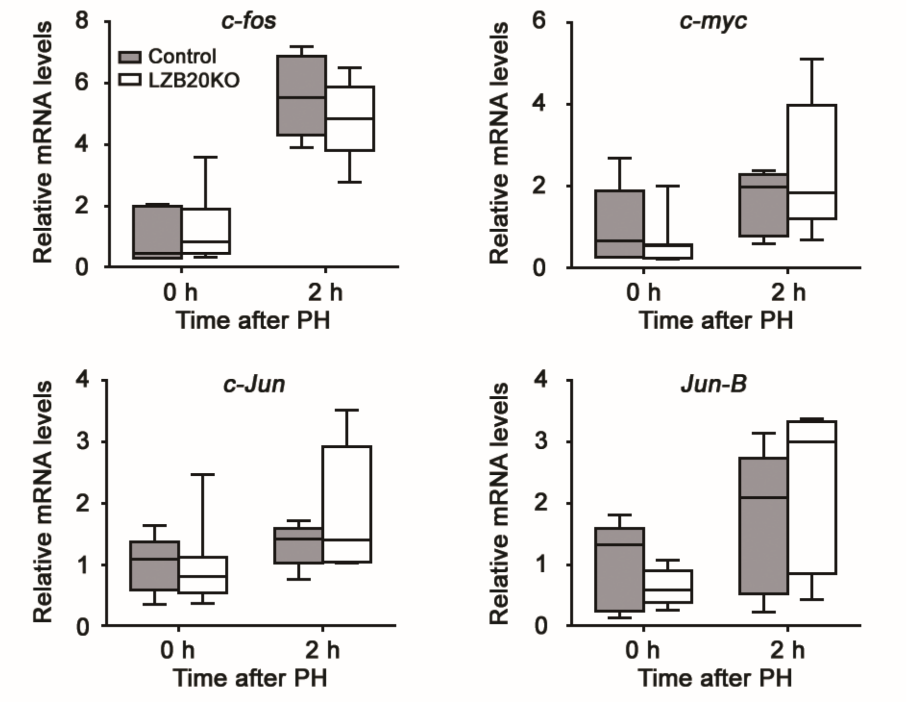

Supplementary Fig.6. **RT-PCR analysis for mRNA expression of immediate early response genes at 2 h after partial hepatectomy**. The data are presented as box-and-whisker plots. n=4.

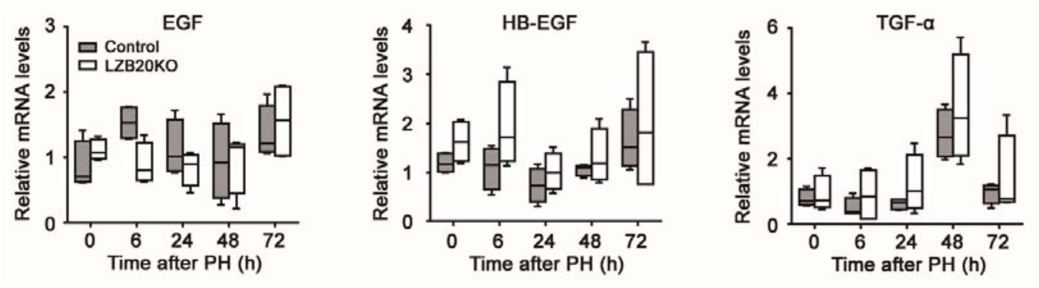

Supplementary Fig.7. **ZBTB20 deficiency does not alter the mRNA expression levels of EGFR ligands in the liver after partial hepatectomy**. The data are presented as box-and-whisker plots. n=4.

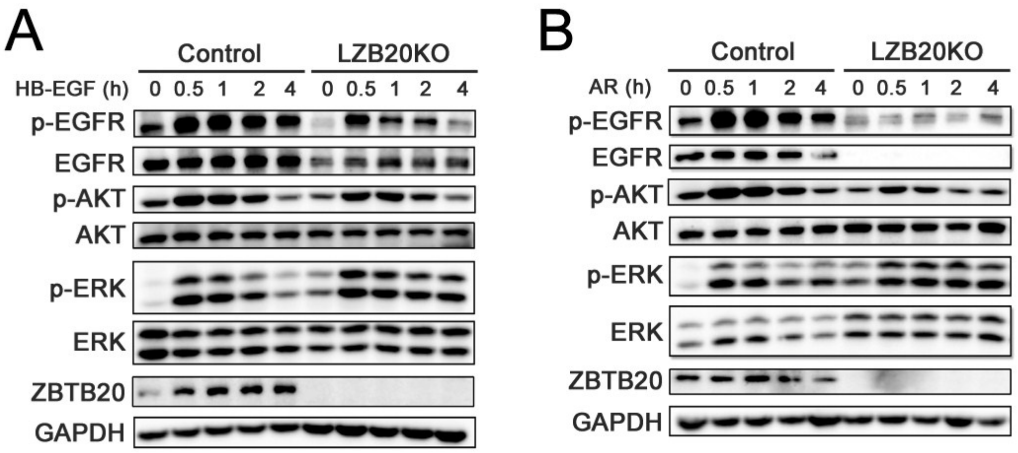

Supplementary Fig.8. **ZBTB20 deficiency attenuates the activation of EGFR and AKT of hepatocytes in response to HB-EGF and amphiregulin**. Primary hepatocytes isolated from control or mutant mice were starved with serum-free media for 12 h prior to the stimulation with 1 ng/ml HB-EGF (A) or 10 ng/ml amphiregulin (B) for indicated time. AR, amphiregulin.

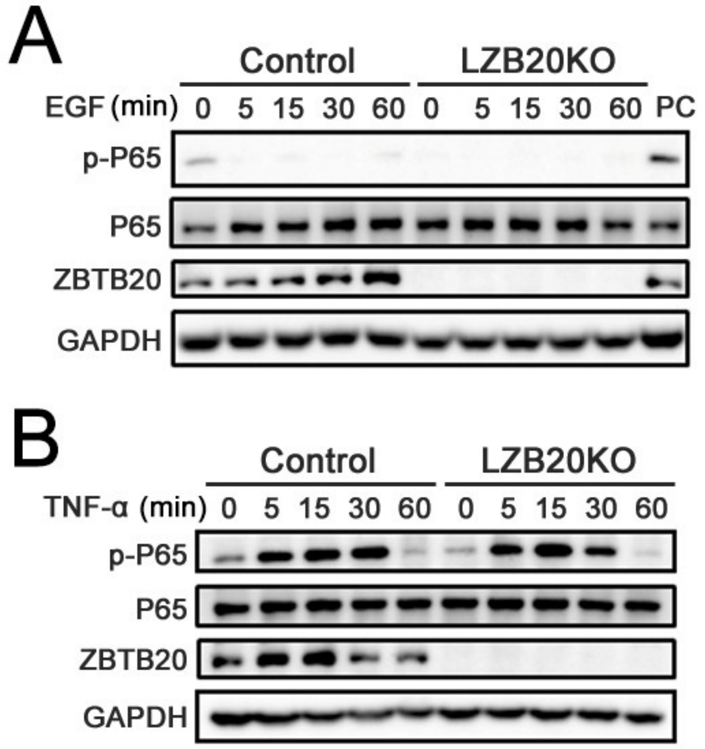

Supplementary Fig.9. **ZBTB20 deficiency does not affect the activation of NF-κB in TNF-α-stimulated hepatocytes**. Primary hepatocytes isolated from control or mutant mice were starved with serum-free media for 12 h prior to the stimulation with 1 ng/ml EGF (A) or 10 ng/ml TNF-α (B) for indicated time. EGF does not activate NF-κB in the hepatocytes. PC, positive control hepatocytes stimulated with TNF-α.

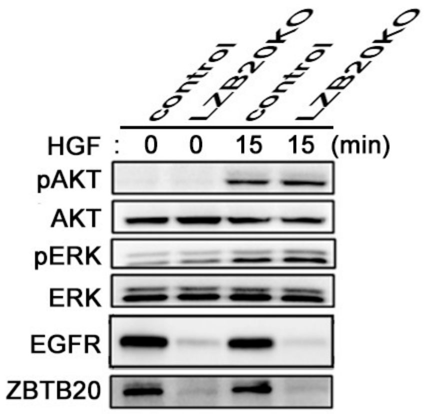

Supplementary Fig.10. **ZBTB20 deficiency does not affect HGF-induced activation of AKT or ERK pathways in hepatocytes**. Primary hepatocytes isolated from control or mutant mice were starved with serum-free media for 12 h prior to the stimulation with 20 ng/ml of HGF for 15 minutes.

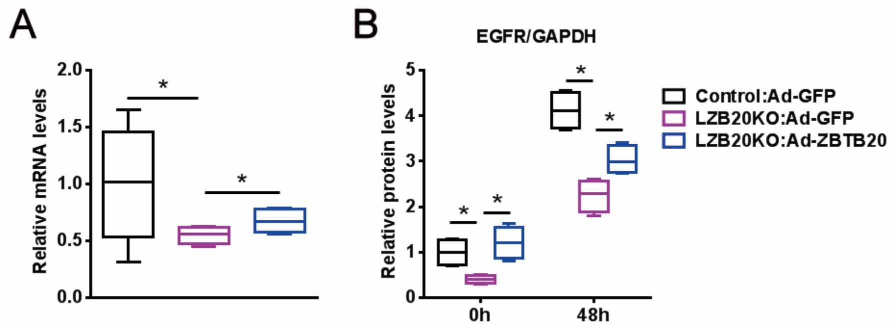

Supplementary Fig.11. **Hepatic ZBTB20 overexpression partially restores EGFR expression in ZBTB20-deficient liver**. LZB20KO and control mice (3~5 months old) were i.v. injected with adenoviruses Ad-GFP or Ad-ZBTB20 (0.1 O.D. per mouse) 14 days prior to PH, and their liver samples were collected at 48 h after surgeries. (**A**) EGFR mRNA expression in the liver was assayed by RT-PCR. (**B**) EGFR protein levels presented in Figure 7A were normalized by GAPDH and relative levels were shown in various groups. The data are presented as box-and-whisker plots. *, $P<0.05$; n=4~6.

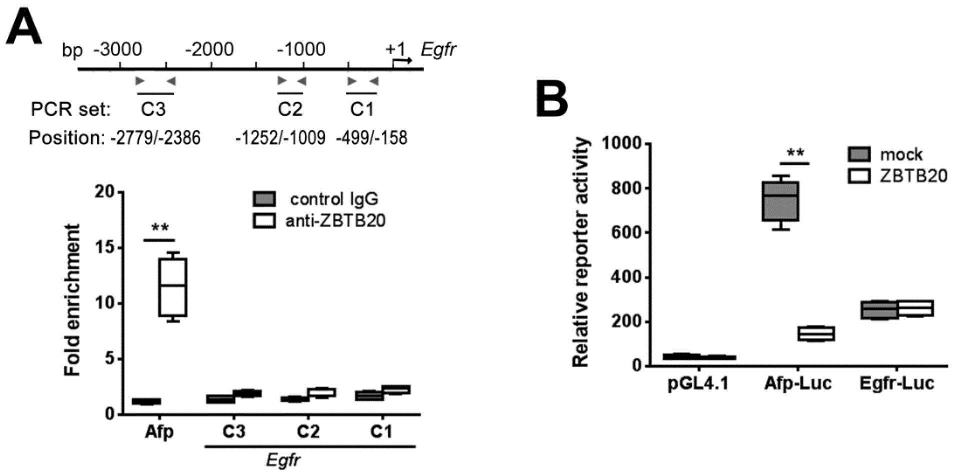

Supplementary Fig.12. **ZBTB20 does not directly regulate the transcription of mouse *Egfr* gene**. (**A**) ChIP analysis shows that ZBTB20 does not bind to mouse *Egfr* gene in the liver. Chromatin was immunoprecipitated with control IgG or anti-ZBTB20 monoclonal antibody 9A10, and subjected to PCR amplification using three sets primers corresponding to the indicated position. The transcription start site of *Egfr* gene is indicated as +1. Afp promoter was detected for ZBTB20 binding as a positive control. (**B**) Reporter assays reveal that ZBTB20 does not regulate the transcriptional activity of mouse *Egfr* promoter. HepG2 cells were cotransfected with the luciferase reporter plasmids driven by Afp (Afp-Luc) or *Egfr* promoter (Egfr-Luc), ZBTB20-expressing plasmids and RL-SV40 as internal control. Relative luciferase activity was standardized with internal control. The data are presented as box-and-whisker plots. n=4. **, P<0.01 vs control (Mann-Whitney test).

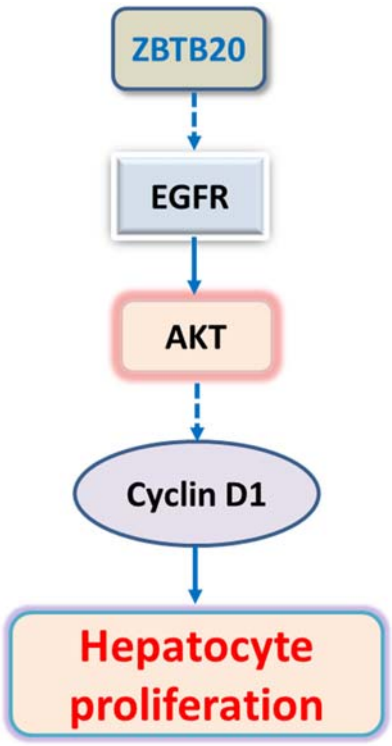

Supplementary Fig.13. Schematic demonstration for the regulation of hepatocyte proliferation in liver regeneration through EGRF expression. ZBTB20 regulates EGFR expression by an indirect mechanism, thereby modulating the activation of EGFR and its downstream AKT signaling pathway, which at least partly contributes to cyclin D1 induction and hepatocyte proliferation in liver regeneration.
